# Parkinson’s patients situation during the SARS CoV-2 pandemic and their interest in telemedicine *A cross-sectional study*

**DOI:** 10.1371/journal.pone.0260317

**Published:** 2021-12-02

**Authors:** Victoria Dorothea Witt, Gabriel Baur, Jule Ecke, Anja Kirchner, Björn Hauptmann

**Affiliations:** 1 Psychiatric Center Rickling, Rickling, Germany; 2 MSH - Medical School Hamburg, Hamburg, Germany; 3 Neurological Center, Segeberger Kliniken, Bad Segeberg, Germany; Universita degli Studi della Campania Luigi Vanvitelli, ITALY

## Abstract

**Background:**

During the SARS CoV-2 pandemic, telemedicine experienced an enormous boom. Also, for Parkinson’s patients there are upcoming alternatives to regular care.

**Objective:**

The aim of the present study was to interview Parkinson’s patients under the impression of the first lockdown in Germany about their health care situation, but especially about the use of and attitudes towards videotherapy and -consultation.

**Methods:**

Northern German members of the German Parkinson Association were mailed a 16-item questionnaire including demographic questions on a one-time basis. The voluntary participants answered regarding their health care situation during the first German SARS CoV-2 lockdown, as well as attitudes towards videotherapy/-consultations.

**Results:**

The 332 (of 974 questionnaires) responding evaluated their care situation predominantly (58.7%) unchanged during lockdown. There was hardly any previous experience in the areas of videotherapy and -consultations (15.4% and 3%, respectively), but at the same time mostly imaginability of implementation (54.2% and 56%, respectively) and the belief that they could motivate themselves to do so (51.8%). A total of 69% welcomed technical support for the implementation of videotherapy.

**Conclusion:**

In principle, there seems to be both, a need and an interest in telematics in healthcare such as videotherapy and video consultations, even if further barriers such as technical implementation need to be addressed. An expansion of telemedical services and infrastructure seems desirable not only in the pandemic situation, but also in the long term against the backdrop of demographic change, especially in an area like Schleswig-Holstein. Further studies are needed.

## Introduction

Therapeutically, Parkinson’s disease as a chronic degenerative disease requires regular care. The reason for this is that typical of the disease mobility, non-motor symptoms and ultimately quality of life are continuously deteriorating [1–3], so that long-term, highly specialized therapies as well as medical consultation and drug adjustments are necessary. Non-drug therapies include, for example, speech therapy, neuropsychology, physiotherapy, occupational therapy or sports therapy, which from a tertiary prevention perspective should ideally be carried out at high frequency and continuously.

In Germany, however, in-patient services (the so-called "Parkinson complex therapy", which is designed to last 3 weeks) are limited in time. Previous outpatient offers can hardly reflect on and provide for the high complexibility in quantitative and qualitative terms. In addition, several different therapies and doctor’s appointments at different locations are difficult to manage for the patients, most of whom are elderly. Due to the chronic progression of the disease with a higher need for care and in view of demographic change with an estimate of over 12 million patients by the year 2040 [4], there have already been initiatives among experts who have dealt with the care situation applying telehealth [5]. Telehealth, respectively telemedicine currently therefore seem highly promising as a modern field of experimentation in which specialists try to improve care and develop concepts to counteract future care bottlenecks. Regarding the situation in Germany, it remains a great challenge in certain regions, such as the federal state of Schleswig-Holstein, to provide specialized care for those affected. In fact, there were and still are concerns about treatments "from a distance". However, there has been relaxation of the ban on remote treatment by the German Medical Association in 2018 [6]. The omens were therefore favorable for an expansion of telemedicine even before the novel coronavirus disease-19. But clearly this development in the entire world, including Germany, has been fueled by the SARS CoV-2 pandemic [7], with professionals exploring different pathways to apply neurological expertise in Parkinson’s disease (e.g. remote neurological consultations, patient-reported outcomes, smartphone applications, etc.) [8], but also in other chronic neurological diseases [9, 10] to ensure the continuity of care. Adoption of telehealth or the entire spectrum of activities used to deliver healthcare at a distance has with the urgent need to reorganize healthcare systems been catalyzed at high pace within the past months [11]. As a result even a digital healthcare revolution even has been proclaimed [12]. With regard to Parkinson’s disease, this gives hope for a further improvement in care, since there has been evidence that telemedicine can connect patients to care, increase access to expertise for patients and providers, and allow more-extensive, less expensive participation in research [13].

Since telemedical care for Parkinson’s in Germany is still in its infancy, the question arose in connection with the pandemic as to how the Parkinson’s patients’ health was doing during the pandemic. Was their care worse and did their subjective general condition deteriorate? Did they continue to be cared for during a passable "lockdown" since worldwide PD management during the pandemic seemed challenging [14]? In this overall context, what is their attitude towards video therapy and medical care via video? There has been evidence for anxiety regarding use of the technical devices in the past [15] and it is also often assumed that older patients in particular could be overwhelmed by the technical requirements for telemedicine.

But is there still a basic willingness to use videotherapy among these patient groups? Videotherapy in this case means a combination of "action observation" and "action execution" therapy [16], which are conveyed to the participant via video format. Among other factors, the possible therapeutic effect is based on the assumption that motor areas are stimulated by mirror neurons during the action [17].

Is the willingness to try videotherapy age dependent? To answer these questions, it seemed appropriate to ask the patients themselves these questions. Aiming to reach as many affected persons as possible in the catchment area of the federal states of Schleswig-Holstein and Hamburg a self-response questionnaire was chosen. Unlike another study on patients view on telemedicine which found high interest (76% of n = 781) in such options [18], we decided to avoid an online survey approach to make the study more inclusive and to avoid bias by primarily reaching in online matters already experienced individuals. In order to reach and question not only technically versed Parkinson’s patients we therefore opted for a postal "analogous" survey.

### Rationale

Objective of this cross sectional-study was to investigate the condition of Parkinson’s patients during the corona pandemic on basis of a comprehensive online self-answered questionnaire. This was done in particular to better assess their health status on the one hand, but also to find out how high the willingness is to use video therapy as a telemedical treatment form in this context.

## Methods

Initially, a 16-item questionnaire was developed as part of a bachelor’s thesis with the support of a Parkinson’s specialist. Subsequently, the questionnaire was tested for comprehensibility and answerability by individual Parkinson patients. The items asked for were related to 6 topics: medical care, burden/affection by the SARS CoV-2 pandemic, change in health status including mental health, maintenance of personal fitness, previous experience with videotherapy/-consultation, openness to videotherapy/-consultation and technical requirements. Possible answers were either summarized in categories (e.g. frequency of physical activity), queried in the form of decision questions or required an evaluation on a Likert-like scale. Questions are presented in detail in Table 1.

**Table 1:** 16 questionnaire items in full text.

Question	Full text
1	Was there a change in the frequency of your medical care (medical and/or therapeutic)?
2	Did you have any difficulties to get in contact with your medical staff (attending doctor, therapist)?
3	How much do you feel burdened by the Covid-19 pandemic and its consequences in general?
4	Did you have to go into quarantine?
5	If you belong to a sports, support or similar group: did you stay in contact with the group members?
6	During this time, have you noticed any deterioration in your physical condition due to Parkinson’s disease (e.g. increased difficulty in performing everyday movements/actions)?
7	During this time, have you noticed a deterioration in your psychological state (e.g. depressive mood)?
8	During this time, did you feel satisfactorily cared for by the health system in Germany?
9	How often were you able to carry out physical exercises (gymnastics, sports) independently as self-training of at least 20–30 minutes duration?
10	Do you already have experience with videotherapy (e.g. physiotherapy, speech therapy, etc.)?
11	Do you already have experience with doctoral videoconsultation?
12	Could you imagine carrying out videotherapy (e.g. physiotherapy, speech therapy, etc.) on your own as an alternative if the outpatient therapy is cancelled?
13	Could you imagine having a videoconsultation with your neurologist as an alternative in case the outpatient practice is cancelled?
14	Would it be possible for you to do videotherapy at home? Do you have enough space and the necessary technical equipment (computer, smartphone, etc.)?
15	Would you welcome technical support in the implementation of videotherapy (e.g. due to lack of technical knowledge)?
16	Do you think you could motivate yourself to do videotherapy regularly?

In addition, demographic data on age, gender, duration of the disease and subjective disease severity were collected. For a better and uniform understanding of the subjects, the term videotherapy was briefly defined on the questionnaire. The definition was inspired by Ertelt et al. [19], but was simplified to improve comprehensibility and to use as little technical language as possible. With regard to ethical aspects this was an anonymous data collection (with no identifying information present, distributed by post through the German Parkinson’s Foundation to their members only), the questionnaire was therefore preceded by an information, respectively consent section, informing participants that the data would be used for research. The return of the completed questionnaire constituted informed consent to act as participant in this research. Since the data was anonymously collected by the German Parkinson’s Foundation under informed consent there was no approval by an ethics committee required. The questionnaire itself was introduced by a request to answer the questions relating to the period between 17.03.2020 and 06.05.2020, in which the measures against the corona virus and its spread were most far-reaching to date in Germany. This period of time was oriented on measures such as the entry stop for third country nationals, a worldwide travel warning, travel restrictions and the closure of many stores classified as non-essential, or the reopening of most stores and the possibility to practice mass and leisure sports under certain conditions. In order to reach Parkinson patients outside of clinical settings, a cooperation with the German Parkinson Association was established. In order to reach as many Parkinson patients as possible within the catchment area of the investigators, all members of the German Parkinson Association in the federal states of Hamburg and Schleswig-Holstein were contacted by mail. A total of 974 questionnaires were sent out. The recruitment, respectively invitation to participate and answer the questionnaire took place between 30.06.2020–17.07.2020. From 18.07.2020 data collection and statistical evaluation for analysis were carried out using IBM SPSS [20]. Sole inclusion criteria for the members of the German Parkinson Foundation were a Parkinson diagnosis determined by a neurologist, residence in the before mentioned catchment area and willingness to complete the questionnaire. Sample size was therefore determined by feasibility. Excluded were members of the German Parkinson Association without a diagnosis of Parkinson’s disease (such as next of kin, health staff, etc.) or Parkinson patients outside of the before mentioned catchment area. Statistical analysis was performed using SPSS Statistics. Internal consistency was evaluated by Cronbach’s alpha coefficient for the above mentioned 16 questions related to six topics. Correlations between metric and nominal scale levels were considered using cross-tabulations (Chi Square Test).

## Results

**Fig 1:**
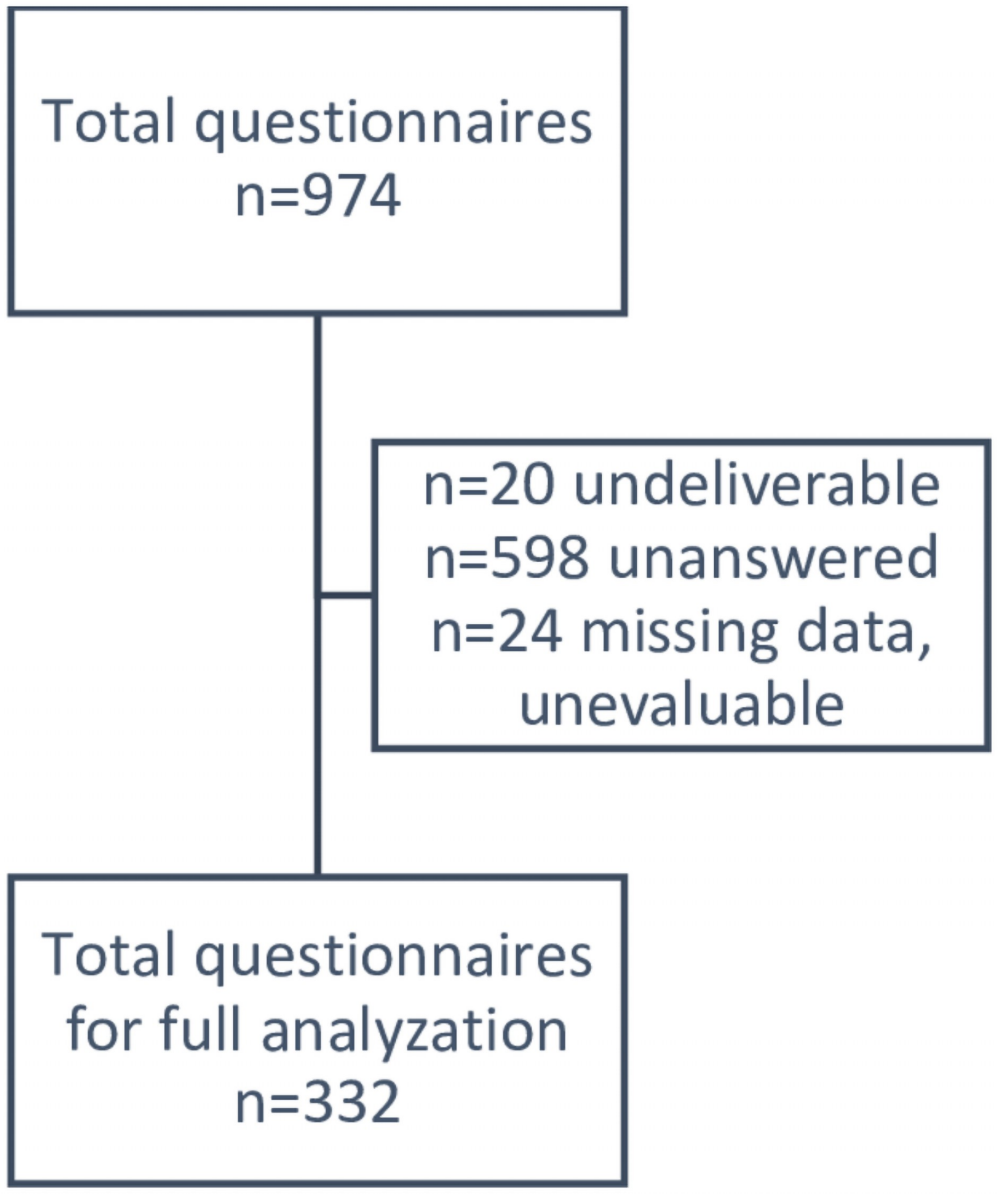
Strobe diagram on total, respectively analyzable questionnaires.

Of a total of 974 postal questionnaires, 332 could be evaluated. A total of 20 questionnaires were returned by the postal service as undeliverable, and 24 questionnaires could not be evaluated due to several missing or inconsistent data (Fig 1).

Demographically, the majority of the participants were male (58%) between 71–80 years of age (42%) and moderately affected (48%) on a self-reporting basis, with the majority having been diagnosed for more than 10 years (48%). The more detailed demographic survey is presented in Table 2.

**Table 2:** Table of demography—Patient characteristics at baseline.

Variables at baseline	*N = 332*
Gender female / male, n (%)	139 (42) / 193 (58)
Age categories in years: n (%)	40–60: 32 (10)
	61–70: 76 (23)
	71–80: 141 (42)
	> 80: 82 (25)
	n/a: 1 (.3)
Years since primary Parkinson’s diagnosis: n (%)	≤ 5: 87 (27)
	6–10: 90 (27)
	>10: 154 (46)
	n/a: 1 (.3)
Severity of Parkinsons’s disease: n (%)	Low: 21 (6)
	Light: 58 (18)
	Medium: 157 (48)
	Heavy: 91 (27)
	n/a: 5 (1.5)

### Medical care

During the period covered by the survey, 195 (58.7%) of Parkinson’s patients experienced no change in the frequency of their medical treatment. A proportion of 71 (21.4%) patients reported that the frequency of treatment had decreased, while subjectively 61 (18.4%) patients reported that the number of treatments actually increased (n/a: n = 5; 1.5%).

At the same time, a clear majority had no problem coming into contact with medical personnel (n = 277; 83.4%), while only 53 respondents (16%) stated that they had contact difficulties (n/a: n = 2; 0.6%). Accordingly, 283 (85.2%) respondents felt that they were satisfactorily cared for by the German health care system and only 43 (13%) indicated that they felt inadequately cared for (n/a: n = 6; 1.8%). Regarding sports and support groups as a possible support tool, 168 (50.6%) stated that they had no further contact with group members, while 79 (23.8%) maintained such contact. It should be noted that 76 respondents (22.9%) did not appear to belong to any sports or support group and stated that the question did not concern them (n/a: n = 9; 2.7%). The internal consistency reliability coefficient (Cronbach’s alpha) for questions related to „medical care”(questions 1,2,5,8) was 0.126.

### Burden/affection by the SARS CoV-2 pandemic

With regard to the Covid-19 pandemic, the average of participants felt moderately affected on a Likert-like scale (x̄ = 4.75; n = 329), with the majority rating 3/10 (17.5%). A total of 11 persons (3.3%) implied a very high level of concern with a rating of 10/10 on a Likert-like scale. At the other end of the spectrum, a total of 28 (8.4%) patients rated 1/10, indicating that they did not feel affected at all (n/a: n = 3; .9%).

Only a very small fraction of the respondents (n = 16; 4.8%) had to undergo conditional quarantine due to Covid-19, while the vast majority was not affected by such measures (n = 314; 94.6%; n/a: n = 2; .6%). For questions related to „burden/affection”(questions 3,4) by the pandemic the internal consistency reliability coefficient (Cronbach’s alpha) was 0.048.

### Change in health status including mental health

In the subjective assessment of a Parkinson-related deterioration in physical condition, the respondents rated themselves on average with x̄ = 4.14 (n = 334) just below the middle on a Likert-like scale. A majority of 76 patients (22.9%) did not find their physical condition impaired at all (1/10 rating), whereas 16 patients (4.8%) found their health severely impaired (10/10 rating; n/a: n = 8; 2.4%). For a subjective worsening of the psychological condition (e.g. depressive episode) a similar result with x̄ = 4.02 (n = 326) was obtained, whilst 79 (23.8%) rated 1/10 lowest and 11 (3.3%) rated 10/10 highest scores on the Likert-like-scale (n/a: n = 6; 1,8%). For questions (questions 6,7) related to „change in health status”the internal consistency reliability coefficient (Cronbach’s alpha) was 0.846.

### Maintenance of personal fitness

A large group of 110 patients (33.1%) were only able to maintain their physical fitness by independent training (e.g. gymnastics, sports) less frequently than once a week. In contrast, 64 (19.3%) reported to do such self-training at least once a week, 92 (27.7%) at least three times a week, 21 (6.8%) at least five times a week and 35 (10.5%) daily (n/a: n = 10; 3%). There was only one question related to “maintainance of personal fitness”, therefore no internal consistency reliability coefficient was calculated.

### Previous experience and openness to videotherapy/-consultation, respectively technical requirements

Previous experience with video therapy (i.e. physiotherapy, speech therapy, etc.) was present in 51 patients (15.4%). The majority of 281 (84.6%) respondents had no previous experience. Even less previous experience existed with medical videoconsultation. A total of 10 respondents (3.0%) had experience with these, while 322 (97%) denied such. Nevertheless, 180 respondents (54.2%) could imagine carrying out videotherapy independently as an alternative in case of failure of the outpatient therapy (n/a: n = 3; .9%). With regard to a medical videoconsultation this was affirmed by 186 (56%; n/a: n = 4; 1.2%). Accordingly, 149 (44.9%) could not imagine videotherapy and 142 (42.8%) could not imagine a medical videoconsultation.

A total of 214 (64.5%) of those surveyed said that videotherapy would be feasible for them. In contrast, a minority of 116 (34.9%) Parkinson patients denied this possibility (n/a: n = 2; .6%). At the same time, on request the majority (n = 229; 69%) welcomed technical support for the implementation of videotherapy and under 1/3 (n = 94; 28.3%) stated that there was no need for it (n/a: n = 9; 2.7%). Over half (n = 172; 51.8%) of the respondents expected to be able to motivate themselves to undergo videotherapy on a regular basis, while just below half (n = 151; 45.5%) did not believe that they could do so (n/a: n = 9; 2.7%). A chi-square test was performed between age group and experience with videoconsultations. No expected cell frequencies were less than 5. In terms of context this resulted in a large effect, though with no significance (χ^2^ = 0.99, p = .80, Cramer’s V = 0.55). For questions related to „previous experience with videotherapy”(questions10,11,14,15,16) Cronbach’s alpha was 0.556. Further on, we performed a chi-square test between age group and imaginability of a videoconsultation as an alternative. No expected cell frequencies were less than 5. There was a significant association between age group and imaginability of a videoconsultation with small to medium effect in terms of context (χ^2^ = 23.78, p = .0, Cramer’s V = 0.27). Internal consistency reliability coefficient (Cronbach’s alpha) for „openness to videotherapy”(questions 12,13) was 0.716.

## Discussion

### Key findings

We analyzed the care situation and successive experiences, or openness to telemedicine and teletherapy of Parkinson’s patients in Germany during the first lockdown of the SARS CoV-2 pandemic as a possible catalyst for this subfield of telematics in healthcare, which is still relatively little used in neurology in Germany.

A worsening of medical care assumed according to our hypotheses did not prove correct from the point of view of the Parkinson’s patients surveyed. 58.7% did not report any changes in the frequency of care, while 18.4% even reported an increase in the frequency of care. Conceivable explanations for the subjectively reported increase could be the facilitated availability of appointments, after the demand for medical services in the general population had decreased [21, 22], or indeed the sporadically observed new introduction of telemedical consultations, respectively teletherapy that was exceptionally in the pandemic situation approved for e.g. physiotherapists. Ultimately, it would be desirable to ask for details on this in future studies, as the point was not addressed in more detail in the context of the current questionnaire. However, a proportion of 21.4% reported a deterioration in medical care, which raises the question of whether this could have been at least partially mitigated by telemedicine services.

The majority of Parkinson’s patients already involved in support groups (50.6%) did not succeed in maintaining contact with the self-help system during the pandemic. Although this would make sense from a psychological perspective, preferably out of a distance e.g. via social media [14], the challenge may have been precisely in the primarily indicated communication via new media. At the same time, virtual support groups appear to be a promising, seminal instrument for connecting, educating and empowering patients [23]. From a providers point of view there seem to be different practices regarding the professional use of social media. Italian neurologists for example seem to often communicate with patients using social media (48%, respectively 56% according to different studies) such as WhatsApp, though a danger that advancement will not go hand in hand with a legal and cultural adaptation and therefore possibly create ambiguity and risks is recognized [24, 25]. To our knowledge although social media does play a role in medical and patient education [26, 27] there are no studies on German neurologists communication with patients through social media since two central hurdles arise from confidentiality and strict (health) data protection [28].

Subjectively on a Likert-like scale from one to ten there was a perceived moderate affection by the pandemic (x̄ = 4.75), moderate worsening of the physical (x̄ = 4.14) and psychological condition (x̄ = 4.02). Reasons in both directions may positively include that “only” 4.8% of participants were personally affected by having to undergo quarantine and the majority received good or even better healthcare during lockdown as discussed above. Negatively it has among the immediate risk of Covid-19 infection been discussed that there has been an emotional burden to the pandemic due to social disconnection causes, aggravation of loneliness, neglect, depression and anxiety, all of which can produce long-term health consequences [29]. In addition, there were indications that patients with other diseases no longer dared to seek treatment, resulting in „collateral damage”[30]. Failure to maintain physical activity is a fear of experts [31] that appears to be at least partially true in the group of respondents, after 33.1% reported exercising less frequently than once a week, although the majority managed to get active on one to several occasions per week. However, there is a lack of comparative figures on the physical activity of respondents prior to the pandemic. With regard to videotherapy and medical videoconsultations, there was little prior experience, although there were slight differences notable. While the clear majority had no experience with medical videoconsultation (97%), 56% could imagine it, whereas videotherapy, with which 84.6% had no experience, an only barely less 54.2% could imagine it for themselves. In comparison with an American study, a similar number of participants showed interest in telemedicine at 52.4% (among n = 1441 participants), with positive associated factors for interest including a long distance from the neurology clinic and transportation difficulties [32]. A 2019 paper also identified benefits of telemedicine such as access to specialists (62%), convenience (60%), or time savings (59%) as part of a patient survey of n = 781 Parkinson’s patients, while disadvantages included lack of hands-on-care (69%), lack of intimacy (43%), and technical difficulties (37%) [18]. A total of 64.5% estimated that videotherapy could be feasible for them and 51.8% that they could motivate themselves to do it regularly. In parallel, 69% would like support in the technical implementation. Basically, there is a positive attitude towards telemedicine in accordance with other studies, but at the same time individual experiences seem among others to depend on technical aspects [33]. From a psychological point of view there have previously been self reported clinical worsenings secondary to social distancing which can be aggravated by a precarious telehealth system which attributes a certain urgency to the implementation of remote multi-professional support. Challenges for the future therefore include the integration of easy-to-use, cost-effective systems that can be practically used by patients as well as embraced within health systems and international professional societies [34]. At the same time, concepts and strategies are needed for those who are not interested in telemedicine services at all, which was 34.9% in our study, or who stated that they could not motivate themselves to undergo videotherapy, which was 45.5% in our study. Since there have been numerous studies on positive and protective effects of physical activity on Parkinson’s disease [35, 36] it seems necessary to further investigate as to why over half of our studies respondents (51.8%) did not expect to be able to motivate themselves to undergo videotherapy on a regular basis. It is to be hoped that, against the background of a possible positive modification of the course of the disease, targeted training offers would be accepted on a broader basis. Especially since delivery via videotherapy could be scalable to a greater extent (e.g., through individual compilation of physiotherapeutic exercises from a kind of video library). As practitioners, we should therefore continue to look at what affects motivation and what might improve it (e.g., music, reward systems, etc.). There appeared to be a large correlation between age group and previous experience with videotherapy, which is not surprising insofar as younger generations are generally more likely to have come into contact with such technology or to have "grown up" with it. At the same time, the age-dependent connection between imaginability of videoconsultations seemed to be rather low to medium, which underlines that telemedicine is possible in all age groups. A paper on the topic that among others reported on successful geriatric telemedicine settings, highlighted once again that the focus of the topic must clearly go beyond simply the provision of equipment and network connectivity, and that a crucial aspect is funding in order for telemedicine to become a mainstream service [37].

### Limitations

In sending the questionnaire by mail or contacting patients generally through the Parkinson’s Foundation we did anticipate potential confounders. It can be assumed that recruitment via the German Parkinson Foundation will lead to some bias. If through the Foundation one reaches anyway rather "engaged" and for new technologies possibly "more open" patients, this applies presumably potentiated for those who would answer the questionnaire in the end. It was also unknown if and to which degree participants had been exposed to new technology as means of therapy before or during the pandemic. Since the survey was conducted under the impression of the SARS CoV-2 pandemic and the associated lockdown, it would be interesting to know whether and to what extent the survey results would have been different "without the pandemic"—the present survey cannot provide any information on this. A further limitation results from the general response rate of the postal survey, after more than 598 questionnaires remained unanswered, which is statistically below the usual approx. 60% mean response rate among mail surveys published in medical journals [38], but may be due to the population of the surveyed chronically ill, often elderly patients. Also, a second reminder or renewed mailing of the questionnaires was omitted in order to obtain a rapid opinion. Furthermore, a selection bias cannot be excluded considering that severely affected persons might have answered less frequently and that only members of the self help community were surveyed, who by their membership show at least some degree of commitment. In order to simplify the answering of the questions, the questionnaire was deliberately kept clear—however, the formation of categories (e.g. with regard to the age structure) has also resulted in limitations regarding the results significancy. The discussion of individual results, e.g. why the state of health subjectively improved during lockdown, is speculative because there was no further information collected in this regard. In principle, supplementary free-text response options would have been helpful, but on the other hand, could have compromised the "quick & easy" completion. With regards to reliability for 3 out of 5 examined topics, Cronbach’s alpha turned out to be significantly below an acceptable value of >0.7. A low internal consistency and measurement with high error variance could therefore be assumed. Ultimately, however, the internal consistency only reflects the correlations of the items, and in the context of categorization, these only matched to a limited extent in terms of content. In addition, the low coefficient is possibly distorted by the predominantly categorical response formats, while interval measurements such as the Likert-like scale are only partially used.

## Conclusions

Overall, our results due to the above limitations may not be representative and must be interpreted with caution. Nonetheless they do show, that there is both a need and an interest for telematics in healthcare such as videotherapy and video consultations, even if further barriers such as technical implementation need to be adressed. While during the SARS CoV-2 pandemic Parkinson’s patients were particularly vulnerable and apparently in some cases at least subjectively undercared for, telematic options seemed particularly indicated, but will as it widely seems also within conventional care carry a value in the future [8, 13]. They could within the pandemic and beyond improve care, promote physical activity, convey hope, and diminish feelings of isolation in Parkinson patients. An expansion of videotherapy/-consultation services and infrastructure seems especially desirable if dwindling resources in the face of rising demand with demographic change are considered, which goes all the more for rural areas such as some regions in the German federal state Schleswig-Holstein. Regarding the generalisability there are and will likely always be patients who are too severely affected from Parkinsons disease or overwhelmed by technical demands and therefore not profit from videotherapy/-consultations. Also, our study indicates that there is a share of patients who are either not interested or does not think they can motivate themselves to participate. It seems therefore from various points of view particularly relevant to consider the patient perspective in assessing the meaningfulness of telemedicine and other remote monitoring. In all these aspects are clearly further studies needed. In regards to this study it would be interesting for further classification and assessment to compare the results with future surveys with similar questions, but in the aftermath of the pandemic, without its influence. Also comparison with findings of other countries, where telehealth projects for Parkinson’s patients may be more established already [5].

## Supporting information

S1 Dataset Study data. (XLSX)

## Abbreviations

PD: Parkinson’s disease
